# An Integrated Dead Reckoning with Cooperative Positioning Solution to Assist GPS NLOS Using Vehicular Communications

**DOI:** 10.3390/s18092895

**Published:** 2018-08-31

**Authors:** Pedro Paulo Liborio Lima do Nascimento, Bruno Yuji Lino Kimura, Daniel Ludovico Guidoni, Leandro Aparecido Villas

**Affiliations:** 1 Institute of Computing, University of Campinas, Campinas 13083-852, Brazil; leandro@ic.unicamp.br; 2 Institute of Science and Technology, Federal University of São Paulo, São José dos Campos 12247-014, Brazil; bruno.kimura@unifesp.br; 3 Department of Computer Science, Federal University of São João Del-Rey, São João Del-Rey 36301-360, Brazil; guidoni@ufsj.edu.br

**Keywords:** Cooperative Positioning, localization, Dead Reckoning, GPS, Intelligent Transportation Systems, Vehicular Ad Hoc Networks

## Abstract

In Intelligent Transportation Systems (ITS), the Vehicular Ad Hoc Networks (VANETs) paradigm based on the WAVE IEEE 802.11p standard is the main alternative for inter-vehicle communications. Recently, many protocols, applications, and services have been developed with a wide range of objectives, ranging from comfort to security. Most of these services rely on location systems and require different levels of accuracy for their full operation. The Global Positioning System (GPS) is an off-the-shelf solution for localization in VANETs and ITS. However, GPS systems present problems regarding inaccuracy and unavailability in dense urban areas, multilevel roads, and tunnels, posing a challenge for protocols, applications, and services that rely on localization. With this motivation, we carried out a characterization of the problems of inaccuracy and unavailability of GPS systems from real datasets, and regions around tunnels were selected. Since the nodes of the vehicular network are endowed with wireless communication, processing and storage capabilities, an integrated Dead Reckoning aided Geometric Dilution of Precision (GDOP)-based Cooperative Positioning solution was developed and evaluated. Leveraging the potential of vehicular sensors, such as odometers, gyroscopes, and digital compasses, vehicles share their positions and kinematics information using vehicular communication to improve their location estimations. With the assistance of a digital map, vehicles adjust the final estimated position using the road geometry. The situations of GPS unavailability characterized in the datasets were reproduced in a simulation environment to validate the proposed localization solution. The simulation results show average gains in Root Mean Square Error (RMSE) between 97% to 98% in comparison with the stand-alone GPS solution, and 83.00% to 88.00% against the GPS and Dead Reckoning (DR) only solution. The average absolute RMSE was reduced to the range of 3 to 5 m by vehicle. In addition, the proposed solution was shown to support 100% of the GPS unavailability zones on the evaluated scenarios.

## 1. Introduction

The advent of large urban centers and the evolution of traffic systems has improved the transportation of people, assets, and services. Consequently, new problems have arisen, such as traffic jams and thus, the need for traffic management and accident prevention. In this context, the Intelligent Transportation Systems (ITS) paradigm aims to provide methods of communication, processing, and data storage to perform traffic management [1,2,3].

Among the several technologies and standards of communications in ITS, the Vehicular Ad Hoc Networks (VANETs) technology and the WAVE 802.11p standard are the main options regarding the capacity of real time processing and instantaneous inter-vehicle communication. In this context, several applications, services, and protocols, ranging from comfort to security, rely on localization for complete functionality. Each of these services rely on some location system and require different levels of accuracy for their full operation [1,4].

The immediate solution for localization is a Global Navigation Satellite System (GNSS), and the most widely deployed GNSS is the Global Positioning System (GPS). However, GPS presents several problems in urban dense areas, vast vegetation zones, multilevel roads, and tunnels. The problem of the multipath effect also occurs and satellite signals are reflected or refracted by buildings and materials in the line-of-sight (LOS). In tunnels, GPS have the worst performance due to the non-line-of-sight (NLOS) with satellites, and the service becomes unavailable [2,4]. These GPS features pose challenges for protocols, applications, and services that rely on localization.

For example, emergency and rescue services such as police, fire-fighters, and ambulances, are unable to find the specific location of an accident in time. In general, there are several levels of accuracy for the different classes of application in VANETs and ITS [1]. For critical applications, localization becomes more essential. Some of these applications control the vehicle for short periods and are highly sensitive to delays greater than 100 ms, with a 20 ms limit for pre-crash sensing [5].

The market of vehicle sensors has increased in previous years with investments from academia and industries. The global market for automobile sensors reached nearly $23.5 billion in 2015, $26.3 billion in 2016, and should reach $43.4 billion by 2021 [6]. Currently, each vehicle has an average of 60–100 sensors on board. Because cars are rapidly getting smarter, the number of sensors is projected to reach as many as 200 sensors per car. These numbers inidicate that approximately 22 billion sensors will be used in the automotive industry per year by 2020 [7]. Therefore, sensors are already off-the-shelf devices in vehicles and will become too much more common in the following years, as shown in recent studies [8].

In this regard, this study relies on low cost sensors (i.e., GPS, gyroscopes, digital compasses, and odometers) and the use of inter-vehicle communications to provide localization and improve accuracy in a cooperative way. Moreover, the proposed integrated Cooperative Positioning (CP) solution provides localization in areas of GPS unavailability such as tunnels. Our contributions are as follows:

- GPS unavailability scenarios were characterized from real GPS datasets as proof of concept.
- A Dead Reckoning (DR) approach was developed to cover GPS unavailability using off-the-shelf sensors.
- An Integrated DR Aided Cooperative Positioning approach using vehicular communication (WAVE IEEE 802.11p) was developed to calibrate Dead Reckoning sensors and improve accuracy in the final positioning. Our approach is only based on Vehicle-To-Vehicle (V2V) communications and uses the Geometric Dilution of Precision (GDOP) concept.
- A Map Adjustment (MA) technique was developed to adjust the final estimated position of the vehicle to enter the road boundaries.

This work is organized as follows. The related work is presented and discussed in Section 2. Section 3 presents the theoretical foundation about localization that is applied in this work. Section 4 presents all concepts involved in the proposed integrated DR aided CP solution. The experimental results are presented in Section 5. Finally, the final remarks and perspectives for future work are discussed in Section 6.

## 2. Related Work

Localization in VANETs and ITS can be divided in two main categories regarding their literature: solutions that apply the vehicular network to assist the GPS system in critical scenarios and solutions that do not consider the GPS system.

In the first category, the purposed solutions [9,10,11,12] combine the information provided by GPS receivers with the kinematic information of vehicles. These works reported an accuracy level in the range of 3 to 5 m for the final vehicle estimation using radio-raging and range-rating techniques, such as the Angle of Arrival (AOA), the Time of Arrival (TOA), the Received Signal Strength Indicator (RSSI), and the Round-Trip Time.

In the second category, to substitute for the GPS system, the proposed solutions apply radio-frequency technologies with or without Road Side Units (RSUs) [13,14,15]. However, the inherent features of inter-vehicle communications, such as the high speed of vehicles, dynamic network density, and short life-time of links, impose several challenges for localization approaches without GPS [11].

In Ref. [9], the authors introduce an algorithm based on two main steps: initialization and refinement. During initialization, vehicles exchange GPS positions through trilateration. During refinement, vehicles use RSSI information to measure distances between them. It is also applied an Extended Kalman Filter technique to couple a vehicle’s kinematics information with GPS information.

In Ref. [15], the authors proposed an algorithm named Relative Distributed Ad-Hoc Localization (RECOP). RECOP uses the distance between vehicles, the Angle of Arrival (AOA) technique, and kinematics information to construct a relative digital map. Vehicles exchange their maps in a clustering process. As a result, each vehicle has an estimative of the position of their neighbors. One disadvantage is that the number of maps exchanged becomes too large as the number of vehicles increases, causing a large overhead in the network. Also, the authors do not consider that the initial position needs to be provided by some localization system as GPS, presenting a risk of outages.

The work proposed by Ref. [16] considers the constant availability of Differential Global Positioning System (DGPS). They proposed an approach where vehicles are used as DGPS base stations and exchange information using IEEE 802.11p radio technology. Simulation results showed a performance of 5 m. In Ref. [12], the authors proposed a distributed approach that enable vehicle fuses, GPS, and radio ranging information using an Extended Kalman Filter to improve their location estimation. The results showed an accuracy of 3 m against 6.6 m of the stand-alone synthetic GPS accuracy.

The use of RSUs in a V2I communication paradigm was studied in Ref. [13]. RSUs provide the initial vehicle position which, after this initialization phase, operates using a Dead Reckoning scheme. The two main disadvantages are the use of many RSUs along the roads, which increase the costs of the system (the average cost of one RSU is about USD 900,000). The evaluation was performed only on one straight road in an open area; therefore, the dead reckoning model used was much simpler than one applied in a more realistic scenario with curves.

In Ref. [14], the authors proposed a GPS-free localization framework that used two-way time of arrival for ranging between vehicles and Dead Reckoning to locate the vehicles based on communication with RSUs in a V2I fashion. Similar to Ref. [13], several RSUs are needed to provide localization along the roads (increasing the total cost of the system). The scenarios evaluated are also straight roads, which makes it much simpler for the kinematics model to perform Dead Reckoning.

A data fusion model to integrate distance through Ultra Wide Band (UWB) radio and GPS information using vehicles and RSUs was proposed by Ref. [17]. However, the authors relied on RSUs to cover the GPS area of unavailability and to improve the localization accuracy inside tunnels. Moreover, a straight road was used to evaluate the proposed solution, which favors the mobility model of vehicles. Final RMSEs in the range of 2 m were achieved. In this work, we propose a different solution to the literature, namely, a robust solution based on Dead Reckoning integrated with Cooperative Positioning using V2V vehicular communications and a digital map based adjustment.

## 3. Theoretical Foundations of Localization

This section describes the main concepts involved in the localization techniques that compound the proposed integrated Dead Reckoning aided Cooperative Positioning solution. The following sections describe vehicle positioning sensors, Dead Reckoning, the received signal strength indicator, and multilateration.

### 3.1. Vehicle Positioning Sensors

In this work, we propose the integration of vehicular vehicular sensors with 802.11p vehicular wireless technology. In this subsection, we describe the sensor models considered to employ the Dead Reckoning technique that is explained in the next section. Nowadays, there are several vehicle kinematic sensors available, such as odometers, accelerometers, gyroscopes, compasses, and others that can be used to estimate the vehicle motion. The main idea is to use these sources of information to assist GPS estimation. Moreover, these sensors are used to cover the GPS unavailability [1,4].

There are three main types of *gyroscopes*: optical fiber gyroscopes, laser ring gyroscopes, and microelectrochemical gyroscopes (MEMS gyroscopes) [7]. The first two have high performance but also have a high manufacturing cost and are mainly used in aircraft, boat, and robot industries. Micro-electromechanical gyroscopes are widely used in off-the-shelf devices, such as smart phones, digital cameras and vehicle navigation systems. A gyroscope has some sources of bias, such as temperature, material waste and so on. A model that describes the output of a gyroscope considering its sources of bias is defined in Equation (1).

(1) $${\hat{\delta }}_{i}=\delta_{i}+\sigma \sqrt{\Delta_{t}}+\beta_{instability}$$

According to [18], these two sources of bias affect the output of the gyroscope sensor. $\delta_{i}$ is the real rate of angular variation. $\sigma \sqrt{\Delta_{t}}$ is a noise represented by a zero mean Gaussian white noise whose standard deviation grows proportionally with the square root of time. This noise is named the Angle Random Walk (ARW) and is given in units $\left({{(}^{\circ }/\sqrt{seg})}^{2}/Hz\right)$. $\beta_{instability}$ is the sensor instability given in degrees, and it grows linearly with the time. ${\hat{\delta }}_{i}$ is the estimated angular variation considering these sources of bias. Constants to these sources are achieved in one process named the Allan Variance analysis [4].

An *odometer* supplies information about the traveled distance of a vehicle by measuring the number of full and fractional rotations of its wheels. This is mainly done by an encoder (a device that outputs an integer number of pulses for each revolution of the wheel) [4]. The number of pulses during a time interval is then periodically mapped to an estimate of the traveled distance. This is performed in each time interval by multiplying the number of pulses by a scale factor, depending on the wheel radius. The odometer information can be easily captured by an On-Board Diagnostics 2 (OBD2) [19] device.

A *digital compass* is an electronic device constructed from magnetometers, that gives the angle (azimuth) of the vehicle in relation to the Earth’s magnetic true north. The integration of the gyroscope output with the angle estimated by the digital compass informs the absolute angle at which the vehicle is traveling, considering a system of coordinates [4].

### 3.2. Dead Reckoning

The process of transforming the measurements from the sensor of the vehicle into an estimated position is called Dead Reckoning (DR). The localization DR technique computes the current position of the vehicle based on its last location estimation. In a local coordinate system, the DR solution is given by trigonometric calculations. Given a system of coordinates (x,y) with origin at (0,0), the DR solution is given by a set of equations described as follows:

(2) $$x_{i}=x_{0}+\sum_{i=0}^{k-1}s_{i}\cos\theta_{i};$$

(3) $$y_{i}=y_{0}+\sum_{i=0}^{k-1}s_{i}\sin\theta_{i}.$$

**Figure 1:**
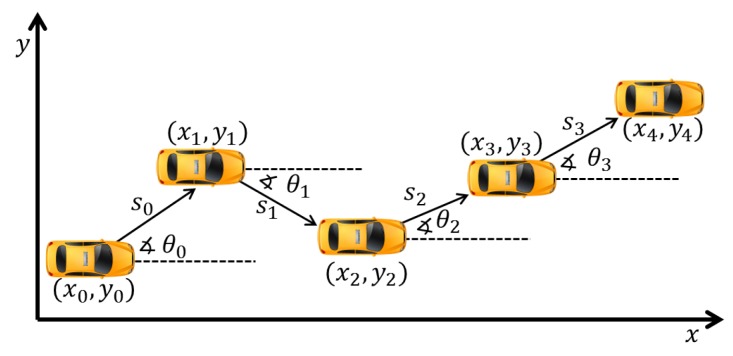
Dead Reckoning.

Here, ($x_{0},y_{0}$) is the initial vehicle location at time $t_{0}$. $s_{i}$ and $\theta_{i}$ at time $t_{i}$ are, respectively, the shortest path and the absolute heading of vehicle in relation to the earlier position $\left(x_{i-1},y_{i-1}\right)$ at time $t_{i-1}$. The relative heading (bearing) is defined as the difference between absolute headings at two consecutive instances and is denoted by ${\hat{\delta }}_{i}$. Given the relative heading measurements, ${\hat{\delta }}_{i}$ in the range of times $t_{0},t_{2},\ldots ,t_{i}$. The absolute heading ($\theta_{i}$) at time $t_{i}$ is computed by Equation (4) (this process is supported by the gyroscope and digital compass). The distance ($s_{i}$) is given by Equation (5) (supported by the odometer). Figure 1 illustrates a vehicle with five different positions $\left(x_{i},y_{i}\right)$, distances $\left(s_{i}\right)$, and absolute angles $\left(\theta_{i}\right)$ over time.

(4) $$\theta_{i}=\sum_{i=0}^{k-1}{\hat{\delta }}_{i}$$

(5) $$s_{i}=\sqrt{\left(x_{i}-x_{i-1}\right)+\left(y_{i}-y_{i-1}\right)}.$$

Algorithm 1 describes the DR technique in detail. Line 1 establishes whether the GPS is available. The loop on lines 2–7 is executed when GPS is unavailable. On line 3, the distance ($s_{i}$) is obtained from the odometer. On line 4, the absolute angle ($\theta_{i}$) is obtained from the estimative ${\hat{\delta }}_{i}$ provided by the gyroscope (Equation (4)). On lines 5 and 6, the coordinates $\left(x_{i},y_{i}\right)$ are obtained from Equations (2) and (3). These steps are repeated during GPS unavailability. 

**Algorithm 1:** Dead Reckoning Algorithm.

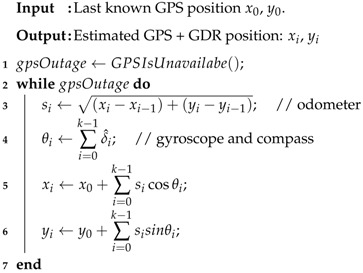



### 3.3. Received Signal Strength Indicator (RSSI)

The Received Signal Strength Indicator (RSSI) radio ranging technique uses the received signal strength to measure the distance between the sender and receiver nodes. Some advantages for the use of RSSI is that it does not need any specialized hardware. On the other hand, depending on the RSSI model, the localization accuracy cannot be so precise [9], mainly because the vehicles travel in different regions with different physical aspects that affects the signal propagation behavior over a short time period. This phenomenon affects the distance estimation achieved from the RSSI model.

Given a power of transmission $\left(P_{TX}\right)$, it is possible to predict the reception power $\left(P_{RX}\right)$. The signal attenuation between the sender and receiver is known as path loss (PL). Regardless of the propagation model, the path loss (in dB) is defined by Equation (6).

(6) $$PL\left(\delta \right)=10log_{10}\left(\frac{P_{TX}}{P_{RX}}\right).$$

Assuming isotropic antennas and a line-of-sight (LOS) between the sender and receiver without obstructions or interferences, the *path loss* is given by Equations (7) and (8).

(7) $$PL_{0}=10log_{10}\left(\frac{16\pi^{2}}{\lambda^{2}}\right)$$

(8) $$PL=PL_{0}+10log_{10}{\left(\delta \right)}^{\alpha }.$$

Here, $\lambda =\frac{c}{f}$ defines the wavelength for one specific frequency (*f*; 5.89 GHz for the center channel (CCH) of 802.11p WIFI technology). The constant c=299,792,458.0 is the speed of light in a vacuum. δ is the distance between the sender and receiver nodes. The constant α determines the signal power decay. Additionally, a random variable with a zero mean and standard deviation $\sigma_{S}$ determines the shadowing effects [20].

Given these points, the RSSI estimation on the receiver is given by Equation (9). This RSSI model is known as *log normal shadowing*. When the effects of shadowing are not considered, the model is known as free-space.

(9) $$RSSI=P_{TX}-PL_{0}+10\alpha log_{10}\delta +S,\;\delta_{min}⩽\delta ⩽\delta_{max}$$

**Figure 2:**
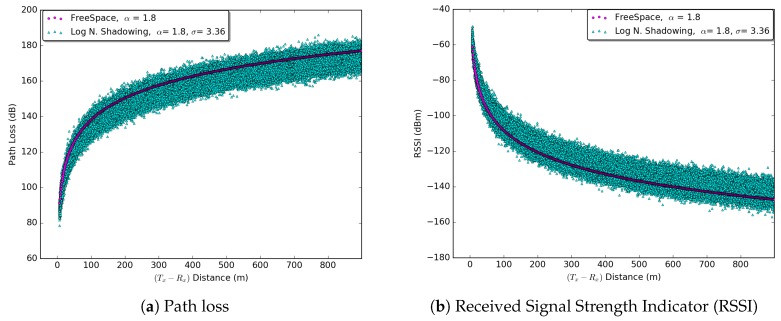
(**a**) Path loss with α and σ values for a high density urban environment [21]. (**b**) RSSI for a transmission power of 20 mW on the WAVE Center Channel (CCH) i.e., 5.80 GHz.

The test-bed results presented in Ref. [21] suggest the values $PL_{0}=53.57$, α=1.77, and $\sigma_{S}=3.36$ for a high density urban vehicular environment with LOS conditions between the sender and receiver. Equation (10) is used to retrieve the distance. Figure 2a shows the increase in path loss as the distance between sender and receiver increases. Figure 2b shows the RSSI behavior (weakening in the signal) as the distance between the sender and receiver increases.

(10) $$\delta =10^{\frac{P_{TX}-RSSI-PL_{0}-S}{10\alpha }}$$

### 3.4. Multilateration

The radio multilateration technique uses geometric concepts and information about the signal propagation model to perform localization. The node that aims to discover its own location (*unknown node*) uses the positions provided by neighbor nodes (*anchor nodes*—nodes with their positions) and ranging information obtained through RSSI or other techniques, such as TOA, TDOA or AOA [11]. The set of coordinates of the *n* anchor nodes, $\left(x_{i},y_{i}\right)$, i=0,1,…,n, are all at a radius ($r_{i}$) of distance from the unknown node (node that needs to estimate its position) of coordinates $\left(x_{u},y_{u}\right)$. Assuming isotropic antennas, Equation (11) is achieved. As a result, considering a set of nodes on the network, the system of Equation (12) determines the position and distance relation between the unknown node and the anchor nodes.

(11) $${\left(x_{i}-x_{u}\right)}^{2}+{\left(y_{i}-y_{u}\right)}^{2}=r_{i}^{2}$$

(12) $$\begin{array}{ccccc} {\left(x_{0}-x_{u}\right)}^{2} & + & {\left(y_{0}-y_{u}\right)}^{2} & = & r_{0}^{2}\\ {\left(x_{1}-x_{u}\right)}^{2} & + & {\left(y_{1}-y_{u}\right)}^{2} & = & r_{1}^{2}\\ \vdots & & \vdots & & \vdots \\ {\left(x_{n}-x_{u}\right)}^{2} & + & {\left(y_{n}-y_{u}\right)}^{2} & = & r_{n}^{2}. \end{array}$$

By subtracting the $n^{th}$ equation from the other n−1 equations and rearranging the system of equations, we have the following linear form (13) in $\left(x_{u},y_{u}\right)$ and the matrix form (14).

(13) $$\begin{array}{ccccccccc} 2\left(x_{n}-x_{0}\right)x_{u} & + & 2\left(y_{n}-y_{0}\right)y_{u} & = & \left(r_{0}^{2}-r_{n}^{2}\right) & + & \left(x_{0}^{2}-x_{n}^{2}\right) & + & \left(y_{0}^{2}-y_{n}^{2}\right)\\ 2\left(x_{n}-x_{1}\right)x_{u} & + & 2\left(y_{n}-y_{1}\right)y_{u} & = & \left(r_{1}^{2}-r_{n}^{2}\right) & + & \left(x_{1}^{2}-x_{n}^{2}\right) & + & \left(y_{1}^{2}-y_{n}^{2}\right)\\ \vdots & & \vdots & & \vdots & & \vdots & & \vdots \\ 2\left(x_{n}-x_{n-1}\right)x_{u} & + & 2\left(y_{n}-y_{n-1}\right)y_{u} & = & \left(r_{n-1}^{2}-r_{n}^{2}\right) & + & \left(x_{n-1}^{2}-x_{n}^{2}\right) & + & \left(y_{n-1}^{2}-y_{n}^{2}\right) \end{array}.$$

(14) $$2\left[\begin{array}{cc} \left(x_{n}-x_{0}\right) & \left(y_{n}-y_{0}\right)\\ \left(x_{n}-x_{1}\right) & \left(y_{n}-y_{1}\right)\\ \vdots & \vdots \\ \left(x_{n}-x_{n-1}\right) & \left(y_{n}-y_{n-1}\right) \end{array}\right]\left[\begin{array}{c} x_{u}\\ y_{u} \end{array}\right]=\left[\begin{array}{ccccc} \left(r_{0}^{2}-r_{n}^{2}\right) & + & \left(x_{0}^{2}-x_{n}^{2}\right) & + & \left(y_{0}^{2}-y_{n}^{2}\right)\\ \left(r_{1}^{2}-r_{n}^{2}\right) & + & \left(x_{1}^{2}-x_{n}^{2}\right) & + & \left(y_{1}^{2}-y_{n}^{2}\right)\\ \vdots & & \vdots & & \vdots \\ \left(r_{n-1}^{2}-r_{n}^{2}\right) & + & \left(x_{n-1}^{2}-x_{n}^{2}\right) & + & \left(y_{n-1}^{2}-y_{n}^{2}\right) \end{array}\right].$$

This overdetermined system of equations has the form Ax=b and several solutions. To solve it, it is possible to use the Least Squares method by QR decomposition [22]. An estimation of the unknown node position with coordinates $\left(x_{u},y_{u}\right)$ is thus achieved.

## 4. Integrated Dead Reckoning Aided Cooperative Positioning

Contrary to the non-cooperative approaches where each node self computes and estimates its own location, Cooperative Positioning aims to show that a cluster of nodes cooperating with each other share their measured localization metrics, such as observed error, location estimates, kinematics, and any contextual information, when available. Therefore, a distributed algorithm can be performed to increase location accuracy of each node in the network [11]. The proposed approach can be summarized in three main steps:

- **Mensuration and Exchange Information Phase**: Vehicles collect two main types of information: kinematic data and ranges from the neighbors in a multihop fashion. Vehicle sensors and a GPS receiver are used to collect and provide information about the speed, orientation, and position of the vehicle. The RSSI technique is used to derive the distance between vehicles. The result is a Range Vector (RV) with all the information from neighbor nodes. The information is sent through WAVE 802.11p technology to nearby vehicles.
- **Localization Phase**: In this phase the unknown node estimates its own location. Each vehicle applies multilateration principles using a matrix with measured distances and positions of neighbor nodes. In addition, in this phase the Geometric Dilution of Precision (GDOP) is considered to optimize the location estimation in the multilateration process. The unknown node uses the location achieved to calibrate the DR estimation at the area of GPS unavailability.
- **Map Adjustment Phase**: Vehicles adjust their final estimated positions to the road using the road geometry information provided by a digital map.

**Figure 3:**
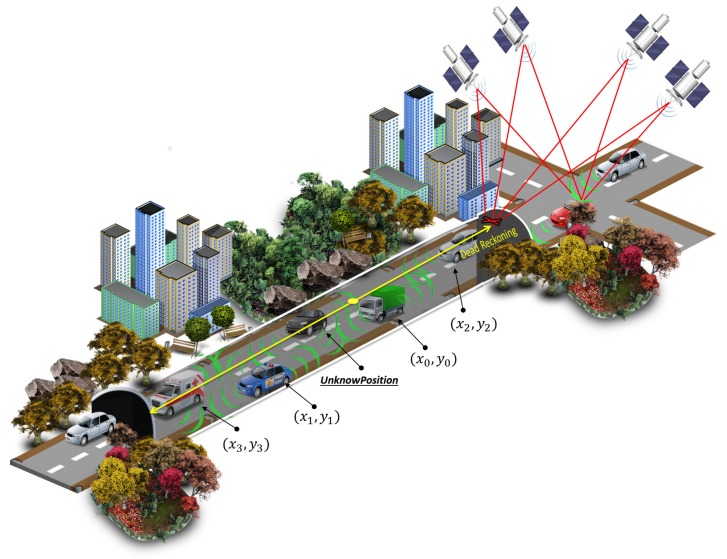
Integrated Cooperative Positioning.

Figure 3 illustrates the application of the proposed integrated Dead Reckoning aided Cooperative Positioning on the area of GPS unavailability. After entering the tunnel, the GPS become unavailable and the vehicle runs the Dead Reckoning with cooperative positioning to estimate its position. The main concepts and approaches developed to optimize the proposed integrated DR aided CP are explained in the next subsections.

### 4.1. Exchange Information Phase

Vehicle exchange kinematics information and position use the beaconing technique and consider a pre-determinated beacon interval.

Once a new beacon sent by the same vehicle is received, the positioning information is updated in a list of anchor nodes. If an anchor node does not send a new beacon until the time limit, this node (identified by the ID) is removed from the list of anchor nodes. This approach is adopted so that the positioning information of neighbor nodes is not delayed. Moreover, if a given anchor node is not more on the communication radius of the unknown node, after the time limit, this information will be erased from the list of anchor nodes.

Algorithm 2 presents a solution to discard old beacons. The maximum time of permanency of an anchor node is given as the input. Line 1 performs the iteration through all nodes on the list of anchor nodes. Line 2 computes one Δ equivalent to the difference between the actual time and the timestamp of the beacon. In line 3, it is verified whether a beacon times out the maximum time of discarding. In affirmative cases, line 4 removes the beacon associated with the respective anchor node. After iterating all anchor nodes, the list of anchor nodes is updated. Note that the time taken to discard one beacon is different from the Time to Live (TTL) associated with the beacon (the TTL by hops is inherent with the multihop approach). 

**Algorithm 2:** Verification and discarding of *beacons*.

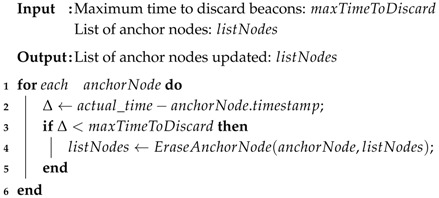



### 4.2. GDOP-Based Positioning Estimation

This work considered the impact of the geometry of nodes on the multilateration process. The proposed algorithm is based on the concept of Geometric Dilution of Precision (GDOP) applied to the selection of GPS satellites. Basically, GDOP is a metric employed to evaluate the influence of satellite geometry on the location estimation of the GPS receiver. More details can be found in Ref. [23].

Assuming the *n* positions $\left(x_{i},y_{i}\right)$ of the anchor nodes, and their respective distances $r_{i}$ for all i=1,…,n nodes on the list of anchor nodes, the multilateration process is applied (Section 3.4) to find the node’s position. After the multilateration process, the estimated distance between the estimated position $\left(x_{u},y_{u}\right)$ and the position of an anchor node of position $\left(x_{i},y_{i}\right)$ is given by $R_{i}=\sqrt{{\left(x_{u}-x_{i}\right)}^{2}+{\left(y_{u}-y_{i}\right)}^{2}}$. The estimated accuracy by multilateration can be statistically described by the covariance matrix which is proportional to the inverse of $G^{T}G$. Here, *G* has the form of Equation (15), and $G^{T}$ is the transpose of *G*. A scalar is used to evaluate the GDOP whose value is provided by Equation (16).

(15) $$G=\left[\begin{array}{cc} \frac{x_{u}-x_{1}}{R_{1}} & \frac{y_{u}-y_{1}}{R_{1}}\\ \frac{x_{u}-x_{2}}{R_{2}} & \frac{y_{u}-y_{2}}{R_{2}}\\ \vdots & \vdots \\ \frac{x_{u}-x_{n}}{R_{n}} & \frac{y_{u}-y_{n}}{R_{n}} \end{array}\right]$$

(16) $$GDOP=\sqrt{trace{\left(G^{T}G\right)}^{-1}}.$$

The GDOP is equivalent to the square root of the trace (sum of the elements on the main diagonal) of ${\left(G^{T}G\right)}^{-1}$. The lower the value of GDOP, the better the geometry of the nodes selected to perform the multilateration is [23]. If there are n>3 nodes, there are different combinations of nodes (with different geometries), and one of these combinations has the lowest GDOP. Therefore, selecting the nodes with the best geometries becomes a combinatorial problem, and the number of combinations is given by Equation (17). It is important to point out the high collinearity of nodes which is constrained by the topology of highways/streets and is inherent in the vehicular networking scenarios, leading to the ill conditioning matrix of the multilateration process [22].

(17) Cnk=n!k!(n−k)!.

**Figure 4:**
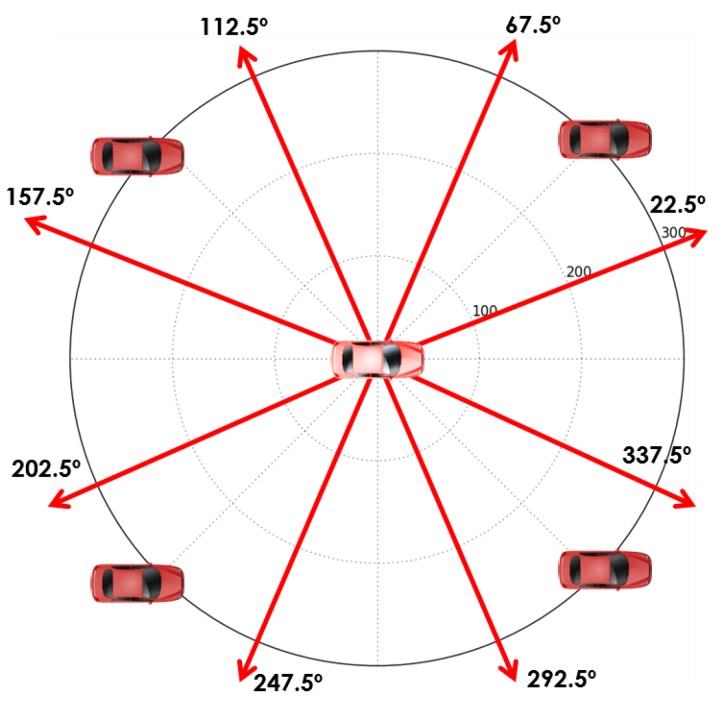
Node selection. In this example, n=4 and k=3.

Given all possible points, we present a node selection solution to decrease the number of combinations required to evaluate the GDOP. The node selection is based on the relative angles observed between the *unknown node* and the *anchor nodes*. As illustrated in Figure 4, these combinations are taken by k=3 (minimum number of nodes to perform one multilateration). This selection follows the same intuition of the GPS satellite selection algorithms, in which the best geometric distribution produces a tetrahedron with the largest volume [23]. In terms of the vehicular network nodes, the best geometric distribution is formed by the nodes with the largest angles between them that are not collinear to the *anchor node*.

Note that node selection occurs in two steps. The first one occurs once a new beacon is received, and it is described in detail in Algorithm 3. If this new beacon comes from one vehicle whose position obeys the angles restrictions and the TTL (measured in number of hops), it is updated on the list of anchor nodes. The second step occurs in the process of Cooperative Positioning (described in detail in Section 4.4), aiming to find the best combination of nodes based on the minimum GDOP value for one multilateration.

Node selection by relative angles can be described as follows (Algorithm 3). On lines 1–4, it is verified whether this beacon is above the TTL by hops. Lines 5 and 6 get the unknown node position and the position of an anchor node inside the beacon message. Lines 7 and 8 compute the relative angle based on the $atan^{2}$ function. Lines 9–12 ensure that the provided position and the computed relative angles respect the angle restrictions. In affirmative cases, the position is updated on the list of anchor nodes. 

**Algorithm 3:** Node selection by relative angles.

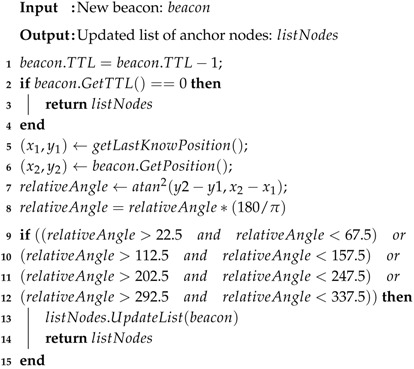



**Figure 5:**
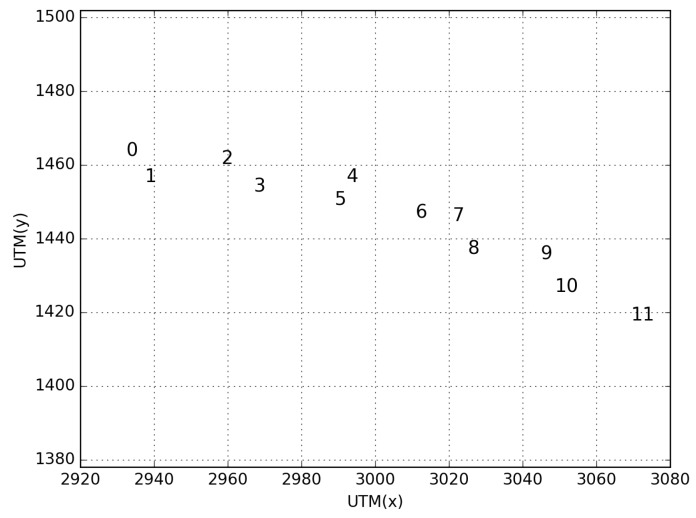
A typical disposition of vehicles inside a road.

In order to clarify the proposed node selection approach, consider the following disposition of nodes in Figure 5 that represents a typical vehicular scenario. Each number represents a different vehicle and its position in the Universal Transverse Mercator (UTM) system of coordinates. In this scenario, we consider vehicle number 3 to be the unknown node. From the perspective of the closest node approach, vehicle 3 considers vehicles 2, 4, and 5. From the perspective of the GDOP (Equation (16)), vehicle 3 chooses the best combination of three vehicles with the lowest GDOP to estimate its position.

Table 1 and Table 2 show the results of the node selection based on the closest nodes and based on the proposed GDOP approach. It is important to note that for each run, the RSSI (Equation (9)) applied to measure the distance from Equation (10), will have a different behavior based on the path loss model (log-normal shadowing in our case). Consequently, the distance between a given pair of nodes may change. The average GDOP value and error for the closest nodes approach are 26.00 and 12.66 m. For the GDOP based approach, the average GDOP and error are 2.16 and 4.31 m. Also, it is possible to note the correlation between the GDOP values and the final error in positioning for each evaluation.

### 4.3. Map Adjustment Using Road Geometry

With the data extracted from a digital map achieved from Open Street Maps [24], a graph was defined from a set of points that compose the roads on the tunnels in the map. These data are in geographic coordinates. Therefore, it was necessary to convert them to the Universal Transverse Mercator (UTM) coordinate system [25] with the support of the *proj4* library [26]. All computations are performed in this coordinate system, since the traffic simulator also uses the UTM coordinate system.

Given a location estimation (x,y) in the UTM coordinate system, the location estimation ($P_{EST}$) is achieved through the map adjustment, as follows. A graph (G(V,E)) was constructed from the information of the digital map, where *V* is the set of vertices and *E* the set of edges. Given each pair of vertices (P,Q)∈E of the graph with coordinates $P(x_{1},y_{1})$, $Q(x_{2},y_{2})$ and a location $P_{EST}\left(x_{3},y_{3}\right)$, the Equation of the line that lies on (P,Q) is defined by Equation (18).

(18) R=P+u(Q−P)

**Figure 6:**
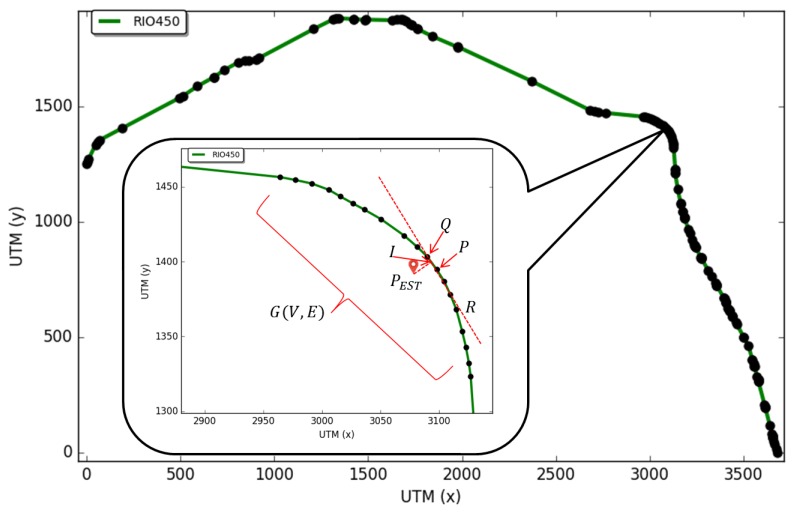
Map adjustment using road geometry.

In Figure 6, the point $P_{EST}$ is the closest to line R exactly on the tangent. The dot product between the tangent and the line is 0, and therefore,

(19) $$\left(P_{EST}-R\right)\cdot \left(Q-P\right)=0.$$

By replacing Equation (19) with Equation (18),

(20) $$[P_{EST}-P-u\left(Q-P\right)]\cdot \left(Q-P\right)=0.$$

By solving Equation (20), Equation (21) is achieved:

(21) $$u=\frac{\left(x_{3}-x_{1}\right)\left(x_{2}-x_{1}\right)+\left(y_{3}-y_{1}\right)\left(y_{2}-y_{1}\right)}{{∥Q-P∥}^{2}}.$$

By replacing *u* in the line equation, the intersection point (I(x,y)) is achieved. Coordinates *x* and *y* are given by Equations (22) and (23):

(22) $$x=x_{1}+u\left(x_{2}-x_{1}\right)$$

(23) $$y=y_{1}+u\left(y_{2}-y_{1}\right).$$

The distance between $P_{EST}$ and line *R* is therefore the distance between $P_{EST}\left(x_{3},y_{3}\right)$ and I(x,y). If the denominator of Equation (21) is equal to 0, *P* and *Q* are the same point, and thus, there is no solution.

If *u* is in the interval (0,1], there is a solution. Otherwise, there is no tangent point in relation to point $P_{EST}$ between points P,Q (inside line *R* in this part). With this approach, the last known position is adjusted to the road geometry, and this process is applied in all points that compose the road in the digital map.

### 4.4. Integrated Cooperative Positioning Algorithm

Algorithm 4 describes the proposed integrated CP solution. This algorithm integrates all concepts and techniques described so far.

Line 1 uses Algorithm 2 to control the beaconing approach and discard old beacons. Line 2 performs Algorithm 3 to select the nodes with the best geometries and reduce the number of node combinations on the GDOP evaluation. Line 3 evaluates the minimum number of nodes required to perform one multilateration (three nodes for 2D localization). Line 4 computes the number of combinations of nodes of size k=3. On line 6, one multilateration is computed and the result of GDOP for this multilateration is evaluated in line 7. Lines 8–11 find the best GDOP with their respective associated positions. If the GPS is unavailable, the position achieved through multilateration is used to calibrate the DR, and the vehicle sensors are reinitialized (lines 14–16). Finally, map adjustment is applied to reduce the estimated position to inside the road boundaries.

### 4.5. Applicability

In this subsection the details about the applicability of the solution in terms of price and how it can be implemented are explained.

The considered sensors are of low cost and off-the-shelf devices. The price of an Inertial Measurement Unit (IMU) device that integrates a gyro and a magnetometer is about $10 USD [27]. The odometer is already integrated in the vehicle and its information can be obtained from the OBD2 [19] vehicle’s port. A digital map can be easily integrated on the vehicle’s On Board Unit (OBU) which has 802.11p technology with storage and processing capabilities.

**Algorithm 4:** Integrated Cooperative Positioning.

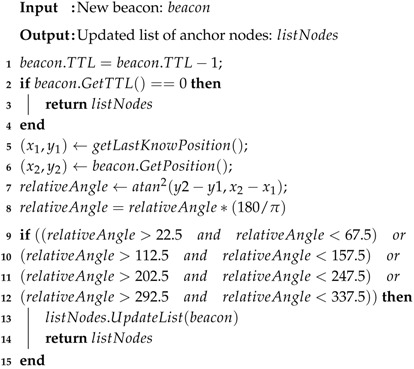



In VANETs, OBU manufacturer provides an OBU unit Software Development Kit (SDK) which enables the development of applications. All the specific details of the proposed solution can be implemented inside the SDK, including the proposed algorithms, network parameters, and integration of sensor data to improve the localization accuracy.

## 5. Simulation Results

This section is divided into four subsections. Section 5.1 presents the results of the characterization of the GPS errors and times of unavailability achieved from real GPS datasets. These situations of GPS unavailability were reproduced in simulations, and the proposed integrated DR aided CP was applied to reduce positioning errors and cover areas of GPS unavailability. Details about the simulation setup are presented in Section 5.2. The positioning results are discussed in Section 5.3, and results concerning network metrics are explained in Section 5.4.

### 5.1. Characterization of GPS Problems from Real GPS Datasets

**Figure 7:**
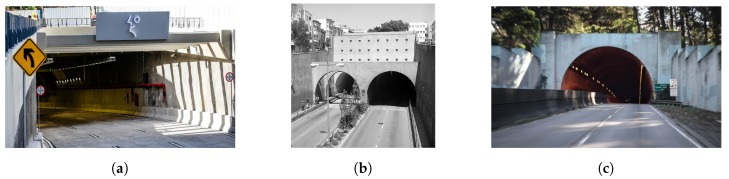
Tunnels which the characterization was performed. (**a**) Rio 450 Years Tunnel [32]; (**b**) Robert C. Levy Tunnel [33]; (**c**) Douglas McArthur Tunnel [34].

In order to identify the GPS unavailability problems, we investigated GPS data from real datasets in our two previous works [28,29]. These data were achieved from routes of taxis and buses of the cities of San Francisco, CA, USA [30] and Rio de Janeiro, RJ, Brazil [31]. Regions of interest near to the Rio 450 Years, Robert C. Levy, and Douglas McArthur tunnels from the cities of Rio de Janeiro and San Francisco, respectively, were selected. While the two first ones are urban tunnels, the last one is placed in a highway. Each tunnel has particular features, such as length, speed limit, number of lanes and so on. Photos of the tunnels are presented in Figure 7, and detailed tunnel features are summarized in Table 3.

Appendix A shows the results of the characterization process. For each outage point on the entrance of the tunnel, there is an another one at the exit, and vice-versa. It is visible that the outages occur not only at the exact entrance and exit of the tunnel, but some meters beforehand in both cases. This is justified by the delay in the process of outage and recovery on the GPS receiver. Additionally, there is a large area of unavailability in which the GPS does not work.

**Figure 8:**
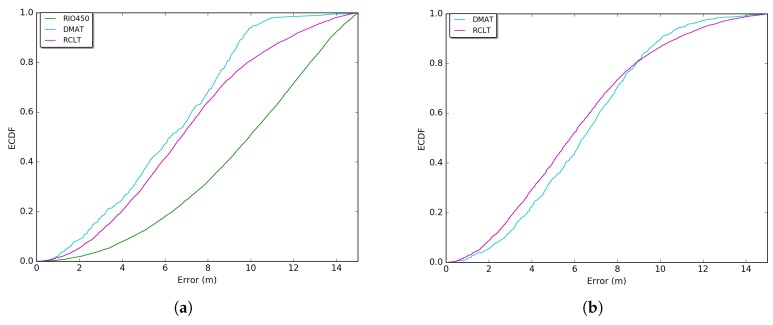
Empirical Cumulative Distribution Function (ECDF) of the real GPS errors retrieved from the datasets. (**a**) GPS errors on the entrance exit sense; (**b**) GPS errors on the exit entrance sense.

**Figure 9:**
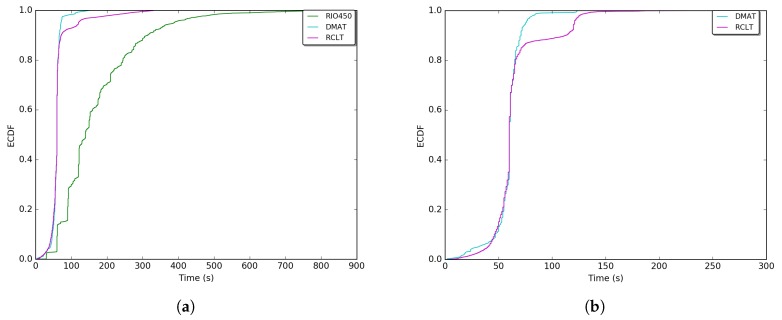
ECDF of the real GPS times of unavailability retrieved from the datasets. (**a**) GPS times of unavailability on the entrance exit sense; (**b**) GPS times of unavailability on the exit entrance sense.

**Figure A3:**
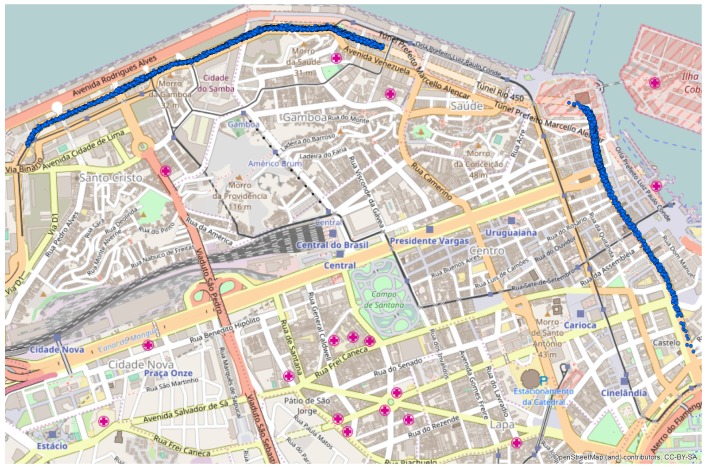
Rio 450 Tunnel, Rio de Janeiro Brasil.

Figure 8 and Figure 9 show the measured GPS errors and times of GPS unavailability through an Empirical Cumulative Distribution Function (ECDF) for each sense of the tunnels. The errors were measured considering the distance between the GPS point and its intersection with the most central lane of the road (at the perpendicular point). The computations were performed considering the Universal Transverse Mercator (UTM) coordinates system. This system of coordinates is used by the SUMO [35] mobility simulator which obtains this information from the OpenstreetMaps *.osm* files [24]. Details about the algorithm to measure the errors can be found in our two previous works [28,29] and is outside the scope of this work. In Figure 8, more frequent GPS errors vary between 5 and 10 m (The RIO450 tunnel is only one way (see Table 3), and for this reason, its error curve does not appear on the exit entrance sense in Figure 8b and Figure 9b. Additionally, a map visualization is provided in Appendix A Figure A3). These errors conform with the errors reported in Refs. [1,4] with a slight reduction. This is due the International GNSS Service (IGS) investments in the deployment of Satellite Based Augmentation Systems (SBAs) around the word [36].

Moreover, considering only the portions with higher frequencies, the most converging times of GPS unavailability for all outages can be evaluated. As shown in Figure 9 for the RIO450, RCLT, and DMAT tunnels, the most representative central values are respectively [98, 219], [52, 63], and [48, 72] s. The distance in outage is directly proportional to the time, and the time and distance are proportional to the tunnel length. These times of unavailability are not sufficient for several applications and services in VANETs and ITS [1,5].

This characterization aims to achieve a real estimation of the GPS error in the region of the tunnels and take information about time and distance that vehicles run without GPS availability. Moreover, with this data, we can reproduce the GPS behavior in simulation environments with better fidelity to the real world.

### 5.2. Simulation Setup

To evaluate the performance of the proposed solutions, we run simulations in the Veins version 4.5 [37] framework that couples the SUMO [35] simulator of mobility version 0.29 and the OMNET++ network simulator version 5.0. With both simulators, it is possible to define all parameters of the mobility of the vehicle and all parameters of the vehicular network following the IEEE 802.11p standard.

The Krauß Model has been configured with 50% driver imperfection [20]. This means that vehicles will have different behaviors regarding acceleration, deceleration, and choice of lanes to drive in. Vehicles were set up to employ speeds varying from the mean and maximum values of the road. The gyroscope model parameters employed on the Dead Reckoning approach were obtained from an automotive gyroscope data-sheet developed by Bosch [27]. The vehicle parameters are summarized in Table 4.

The network simulation parameters are defined in Table 5. The vehicles were configured with a power transmission of 20 mW, enabling a communication range of about 300 m. The nodes of the network disseminated beacons at a frequency of 10 Hz. All beacons were sent through the control channel (CCH) at a rate of 6 Mbits/s. The *log normal shadowing* path loss model was configured in accordance with values of real experiments reported in Ref. [21], under a sensitivity threshold of −84.39 dBm. In other words, beacons with RSSI values smaller than this threshold were not processed. Furthermore, only vehicles outside the tunnel disseminated beacons to cooperate with vehicles under GPS unavailability.

### 5.3. Positioning Results

**Figure 10:**
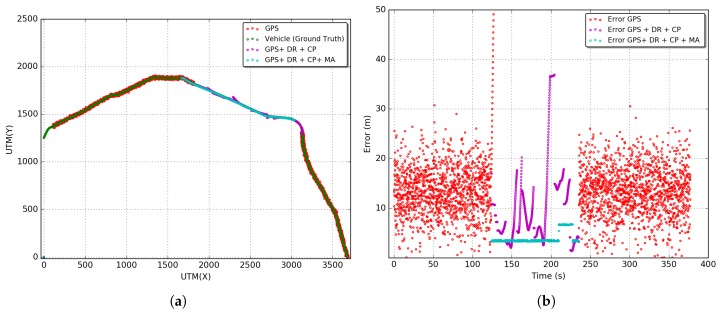
Estimated vehicle positioning and error over time: Rio 450 Tunnel (RIO450). (**a**) Estimated vehicle position; (**b**) positioning error over time.

**Figure 11:**
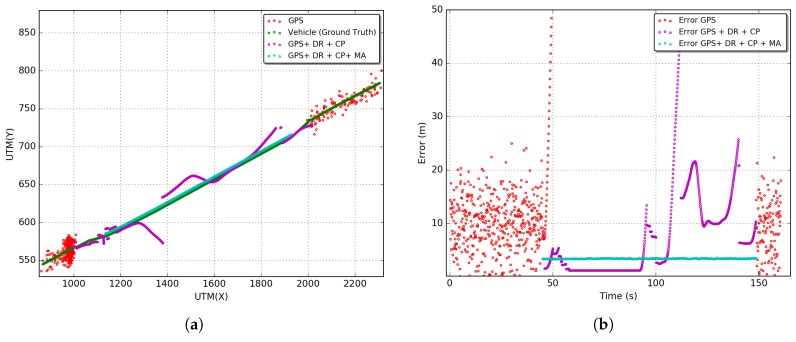
Estimated vehicle positioning and error over time: Robert C. Levy Tunnel (RCLT). (**a**) Estimated vehicle position; (**b**) positioning error over time.

**Figure 12:**
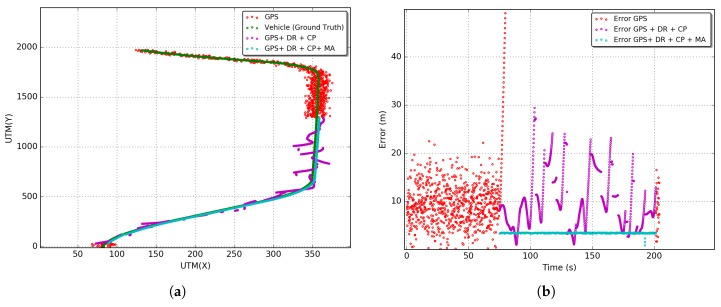
Estimated vehicle positioning and error over time: Douglas Mc. Arthur Tunnel (DMAT). (**a**) Estimated vehicle position; (**b**) positioning error over time.

All results presented in this section were obtained from 33 simulations following a Student’s T-Distribution with a 95% of confidence interval. Figure 10, Figure 11 and Figure 12 show the results of the GPS outages reproduced in the simulation environment with the application of the proposed CP solution. Arbitrary outages were selected from each tunnel characterized in Section 5.1. The main objective was to evaluate the impact of each scenario on the proposed CP solution. Results of trajectory and positioning error over time are presented.

Lines in red, green, magenta, and cyan represent, respectively, the trajectories and errors of the stand-alone GPS solution (GPS), GPS integrated with DR and their sensors (GPS + DR), GPS integrated with DR and CP (GPS + DR + CP) and the last solution with the Map Adjustment (GPS + DR + CP + MA). It is possible to observe that gyroscope random walk effect has a major contribution to the discrepancy in the DR estimative in the outage stage. In general, increasing the time of the use of DR without any source of reinitialization or calibration of sensors leads to a fast evolution of positioning error.

Furthermore, in Figure 10a, Figure 11a and Figure 12a, the DR trajectories are far closer to the vehicle positioning due to the reinitialization/calibration of the sensors using the proposed CP positioning. In Figure 12b of the Robert C. Levy Tunnel (RCLT), errors are under 10 m for 50% of the unavailability time and about 20 m for the other half of unavailability time. The error reaches the peak of 40 m at around 110 s of the trajectory, but it is corrected with the application of CP. In Figure 12b, errors are also maintained below 10 m most of the time, and the error only reaches a peak of 20 m close to the end of the unavailability stage. This is due to the length of this tunnel. In Figure 12b of the Douglas Mc Arthur Tunnel (DMAT), it is possible to see a large path of GPS unavailability where the CP reinitializes the sensors several times, keeping the error bellow 10 m in the first half of the unavailability stage. In the other half, the error fluctuates between 10 and 20 m. In general, we can observe an increase in the error close to the center of the trajectory during the unavailability stage.

After applying the Map Adjustment, the estimated trajectory is even closer to the vehicle trajectory. We can observe errors in the range of 5 to 7 m in the RIO 450 tunnel, and about 3 m in the RCLT and DMAT tunnels. However, note that the reliability of this adjustment depends of the accuracy of the digital map and quality of the estimated position under GPS unavailability. Aiming to obtain the overall behavior of the proposed integrated CP solution, we performed an exploratory analysis using the RMSE (Root Mean Square Error) metric (Equation (24)). This metric is commonly used in the literature to evaluate the error of the localization solutions.

(24) $$RMSE=\frac{1}{n}*\sum_{i=0}^{n}\sqrt{{\left(x_{i-veh}-x_{i-est}\right)}^{2}+{\left(y_{i-veh}-y_{i-est}\right)}^{2}}$$

The variables $\left(x_{i-veh},y_{i-veh}\right)$ and $\left(x_{i-est},y_{i-est}\right)$ are the positions of the vehicle and the estimated positions of GPS, GPS + DR, GPS + DR + CP or GPS + DR + CP + MA. To show the impact of time on the error of the solution, we divide the outage times of each vehicle into levels of GPS unavailability times. Times ranging from 10% to 100% of the total GPS downtime were related to the RMSE.

**Figure 13:**
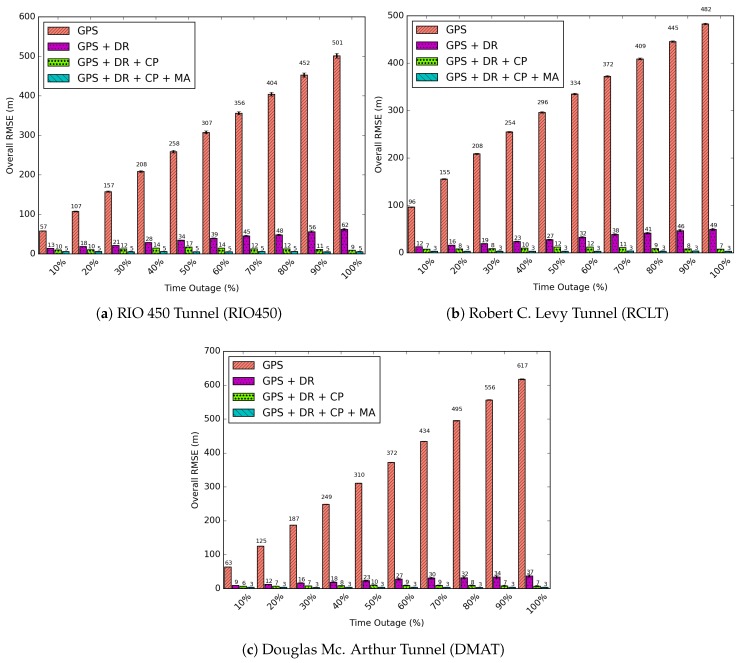
Different percentages of time of GPS unavailability for each evaluated scenario.

In Figure 13 the RMSE for all outages reproduced in the simulation environment are presented. Considering the first 10% of GPS unavailability time, the solutions for GPS, GPS + DR, GPS + DR + CP, GPS + DR + CP + MA achieve, respectively, RMSEs of 57, 13, 10, and 5 m for the RIO450 tunnel; 96, 12, 7, and 3 m for the RCLT tunnel; and 63, 9, 6, and 3 m for the DMAT tunnel. On the other levels (percentages) of unavailability, the GPS and DR solutions increase in accordance with the unavailability time. However, the GPS + DR + CP solution increases up to about 50% at the time of outage and decreases shortly thereafter. This is because only vehicles outside the tunnel disseminate beacons. Consequently, the error on the central region of GPS unavailability is higher than on the tunnel entrances. The maximum values of RMSEs are 17, 12 and 10 m for the RIO450, RCLT, and DMAT tunnels, respectively. The GPS + DR + CP + MA solution maintains the RMSE constant for all outages reproduced in the simulation environment, achieving RMSEs of 5, 3, and 3 m for the RIO450, RCLT, and DMAT tunnels.

To these results, we applied a metric named the average gain. This metric measures how better one solution is than the others in terms of RMSE. The average gain is defined in Equation (25):

(25) $$Average\_Gain=\frac{1}{10}*\sum_{t=10(\%)}^{100\%}\left(\left(1-\frac{RMSE_{S_{1}}\left(t\right)}{RMSE_{S_{2}}\left(t\right)}\right)*100\right)$$

where $S_{1}$ represents the GPS + DR + CP or GPS + DR + CP + MA solutions, and $S_{2}$ represents the other compared solutions: GPS, GPS + DR and GPS + DR + CP. In summary, the gain represents the effective decrease in RMSE applying the integration of the developed solutions.

In detail, the results in Table 6 show that the solutions covered 100% of the unavailable area. The GPS + DR + CP solution achieved average gains between 93% and 98% in relation to the GPS stand-alone and 58% e 63% in comparison with the GPS + DR solution. Moreover, after applying the Map Adjustment (GPS + DR + CP + MA), the gains were notably more effective: between 97% and 98% in comparison with GPS, 83% to 88% against GPS + DR, and 59% to 67% of RMSE reduction in relation to GPS + DR + CP.

**Figure 14:**
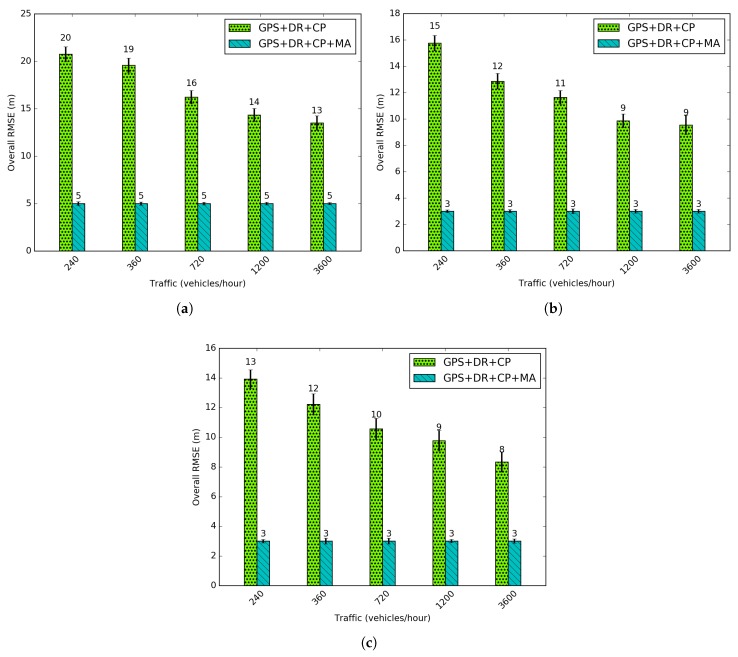
Root mean square errors (RMSEs) for different densities. (**a**) RIO 450 Tunnel (RIO450); (**b**) Robert C. Levy Tunnel (RCLT); (**c**) Douglas McArthur Tunnel (DMAT).

Figure 14 shows the average RMSEs for different traffic regimes. Traffic varies from 240 vehicles per hour (veh/h) to 3600 veh/h which represents the sparsest to the densest network. A decrease in the RMSE is observed with an increase in density. Moreover, after adding a large number of vehicles a low variation in RMSE occurs. From this, we can confirm the fact that the geometry of selected nodes to perform CP (important part of the integrated solution) imposes more influence on the positioning accuracy than the increase in the number of nodes, as previously introduced in Section 4.2. A higher density occurs in the major number of neighbors for one given vehicle. In this sense, a more accurate geometry with the lowest GDOP can be selected, and a better accuracy can be reached. The accuracy using Map Adjustment is the same for all densities, because the geometry of the road is applied to reduce positioning uncertainty.

### 5.4. Network Results

**Figure 15:**
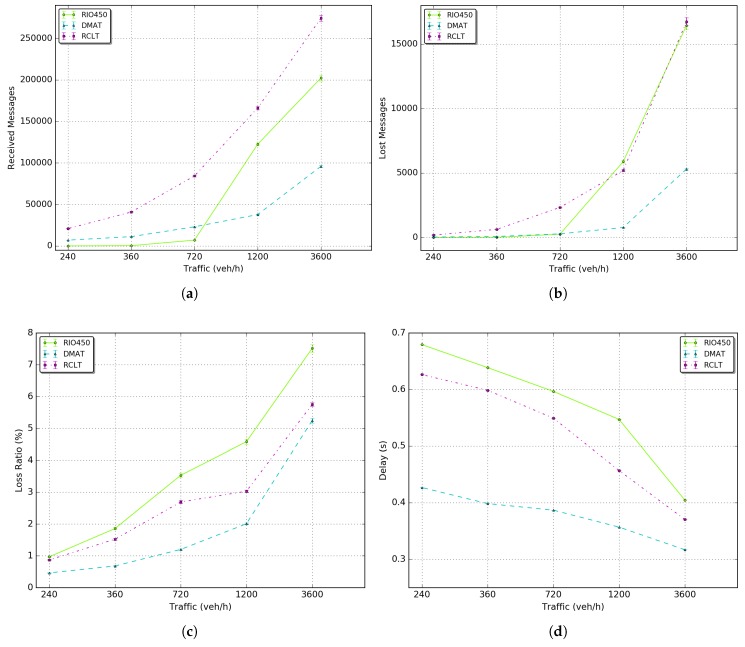
Network metrics: (**a**) received messages; (**b**) lost Messages; (**c**) loss ratio; (**d**) delay.

Figure 15 shows the average results of network metrics by vehicle in each scenario. These results were also obtained following the simulation setup explained in Section 5.2.

Figure 15a shows the average number of received messages that increases as the density of vehicles increases. In general, the number of received messages also increases as the distance and time of the trip increases. This is because more messages are sent and consequently, received by the neighbor nodes. The DMAT tunnel has the lowest number of sent messages because its tunnel size is the smallest. The Rio450 tunnel has a major increasing rate as the density grows. However, the RCLT tunnel has the largest number of sent messages due to the large number of traffic lights in the tunnel region. As a result, vehicles spend more time stopped and consequently, send more beacons over time.

Figure 15b shows the number of lost messages that, for all tunnels, increases as the density increases. In the simulations, beacons are lost by three main factors that are reported by the MAC layer:

- A packet was not received due to bit errors;
- A packet was not received because a packet was being sent while receiving another packet;
- A packet was dropped by the physical layer.

By comparing these results with the ones in Figure 15a, we see that the number of lost messages floated between 1% to 7% for all scenarios. Moreover, despite the number of sent messages of tunnel RCLT being greater than the RIO450 Tunnel, the loss in the RIO450 Tunnel was higher. These details can be better visualized in Figure 15c, which was computed using Equation (26). This behavior occurs because the tunnel RIO450 has the largest extension which represents a major area of GPS unavailability and major effort of CP integrated with DR to compute positions using beacons in a multihop fashion. The DMAT has the lowest loss ratio because its has the smallest area of unavailability and consequently, requires a minor effort to perform the proposed CP solution.

(26) PacketLossRatio=lost_messages/(received_messages+lost_messages)

Figure 15d presents the average delay per message received by vehicle. Is worth noticing that the delay decreases as the density increases. This is due to the fact that, as the density increases, so does the chance of more vehicles receive beacons [2]. DMAT tunnel has the lowest delay, because the lowest size of tunnel, and consequently the minor vehicles inter-distances followed by RCLT and RIO450 tunnel (largest tunnel). These results can be also justified by the fact that for one same network path, as the density increases, one same receiver can be reached with a lower number of hops.

## 6. Conclusions and Future Work

In the last years, several services have been developed to enable Intelligent Transportation Systems (ITS). In this context, several protocols and applications have applied Vehicular Ad Hoc Networks (VANETs) and the WAVE IEEE 802.11p standard as a paradigm of communication between vehicles. These services relies on localization with different levels of accuracy. Thus, in this work, we carried out research on the problems of GPS unavailability caused by Non Line of Sight (NLOS) in tunnels. The immediate solution for localization is the use of a Global Navigation Satellite System (GNSS), such as the Global Positioning System (GPS). However, GNSS systems have inaccuracy and unavailability problems in dense urban areas, vast vegetation zones and multilevel roads. In tunnel scenarios, GPS receivers lose their Line of Sight (LOS) with the satellites and thus, the GPS service becomes unavailable.

Given these considerations, in this work, we presented the development and evaluation of an integrated localization solution considering the potential of vehicular networks. Bearing in mind that sensors (i.e., odometers, gyroscopes, and digital compass) are embedded in vehicles, the developed approach merges a Dead Reckoning (DR) and a Geometric Dilution Of Precision (GDOP) based on Cooperative Positioning (CP) techniques. We developed a Dead Reckoning solution and simulation results (evaluated in tunnel scenarios) showed the viability of the Dead Reckoning solution in the first seconds of GPS system unavailability. The results also show average gains of 60% to 80% in the RMSE when compared to the results of the stand-alone GPS solution.

By using the communication and processing capacities of inter-vehicle communications, we developed a Cooperative Positioning Solution using a multihop V2V communication fashion to periodically reinitialize Dead Reckoning. Moreover, a Map Adjustment technique was developed in order to reduce the final estimated position to the inside road boundaries. The simulation results showed average gains in RMSE of between 97% to 98% in comparison with the stand-alone GPS solution, and 83.00% to 88.00% against the GPS and DR only solutions. In addition, the proposed solution was shown to support 100% of GPS unavailability zones on the evaluated scenarios. The average absolute RMSE by vehicle was reduced to the range of 3 to 5 m.

Future work will comprise different network technologies and use infrastructures such as Road Side Units or Cellular Base Stations, only when they are available. More advanced prediction models using Bayesian statistics are also envisioned to improve the final position estimation.

## Figures and Tables

**Table 1:** Closest nodes approach.

Run N°	Unknown Node	Selected Anchor Nodes	Ref. Pos. (x,y)	Est. Pos. (x,y)	GDOP	Estimated Error (m)
0	3	(2, 4, 5)	(2966.94, 1452.68)	(2970.65, 1459.56)	4.60	7.81
1	3	(2, 4, 5)	(2966.94, 1452.68)	(2958.05, 1419.35)	117.73	34.49
2	3	(2, 4, 5)	(2966.94, 1452.68)	(2965.67, 1450.61)	7.53	2.42
3	3	(2, 4, 5)	(2966.94, 1452.68)	(2964.73, 1441.58)	16.02	11.31
4	3	(2, 4, 5)	(2966.94, 1452.68)	(2971.60, 1461.61)	4.81	10.07
5	3	(2, 4, 5)	(2966.94, 1452.68)	(2969.73, 1462.16)	5.29	9.88

**Table 2:** Geometric Dilution of Precision (GDOP) approach.

Run N°	Unknown Node	Selected Anchor Nodes	Ref. Pos. (x,y)	Est. Pos. (x,y)	GDOP	Estimated Error (m)
0	3	(1, 2, 10)	(2966.94, 1452.68)	(2960.24, 1454.78)	1.57	7.01
1	3	(0, 2, 11)	(2966.94, 1452.68)	(2964.57, 1448.68)	2.43	4.65
2	3	(0, 2, 10)	(2966.94, 1452.68)	(2967.14, 1448.14)	2.85	4.54
3	3	(1, 2, 10)	(2966.94, 1452.68)	(2964.44, 1450.29)	2.00	3.45
4	3	(1, 2, 10)	(2966.94, 1452.68)	(2965.36, 1452.11)	2.16	1.67
5	3	(1, 2, 7)	(2966.94, 1452.68)	(2963.19, 1450.07)	1.96	4.56

**Table 3:** Tunnel technical features.

Name	Type	Dist. (m)	Ways	Lanes	Speed. (km/h)	Acronym
Rio 450 Years	Urban	1480	1	3	60.00	Rio450
Robert C. Levy	Urban	600	2	2	64.00	RCLT
Douglas McArthur	Highway	400	2	2	72.40	DMAT

**Table 4:** Mobility parameters.

Parameter	Value
Mobility Model	*Krauß Model*
Vehicle Length	3.5 (m)
Acceleration	1.0 (m/s${}^{2})$
Speed	RIO 450 = [8.30, 16.70] (m/s)
	RCLT = [8.90, 17.78] (m/s)
	DMAT = [10.00, 20.00] (m/s)
Imperfection	0.5
GPS Error Mean (μ)	RIO450=9.50
	RCLT=6.97
	DMAT=6.19
GPS Error Std. Dev. (1σ)	RIO450=1.14
	RCLT = 1.10
	DMAT = 0.94
GPS Frequency	10 Hz
Gyroscope ARW	$0.063245^{\circ }/s/sqrt$(Hz) [27]
Gyroscope Sensitivity	±2% [27]
Gyroscope Frequency	10 Hz [27]

**Table 5:** Vehicular network parameters.

Parameter	Value
WIFI Technology	WAVE IEEE 802.11p
Frequency	5.89 GHz
WAVE Channel	Center Channel (CCH)
Potency of Transmission ($P_{TX}$)	20 mW
Communication Range	300 m
Transmission Rate	6 Mbit/s
Path Loss Model	lognormalshadowing
Path Loss Parameters	$PL_{0}$ = 53.57 dB, α = 1.77 dB $\;\sigma_{S}$ = 3.36 dB [21]
Sensitivity Threshold	−84.39 dBm
Communication Pattern	V2V
Frequency of Transmission	10 Hz
N° Max Hops	4
Vehicles Traffic	1200 (Veh/h)

**Table 6:** Average gains for GPS + DR (Dead Reckoning) + CP (Cooperative Positioning) and GPS + DR + CP + MA (Map Adjustment).

	*GPS + DR + CP*		*GPS + DR + CP+MA*		
Tunnel	GPS	GPS + DR	GPS	GPS + DR	GPS + DR+CP
RIO450	93.46%	58.04%	97.27%	83.00%	59.00%
RCLT	96.18%	63.43%	98.76%	88.28%	67.87%
DMAT	96.32%	59.53%	98.59%	85.01%	63.66%

**Figure A1:**
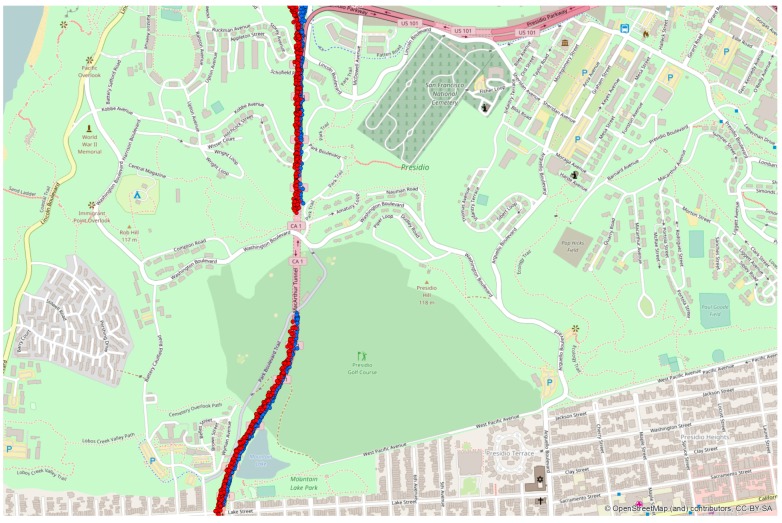
Douglas Mc Arthur Tunnel, São Francisco USA.

**Figure A2:**
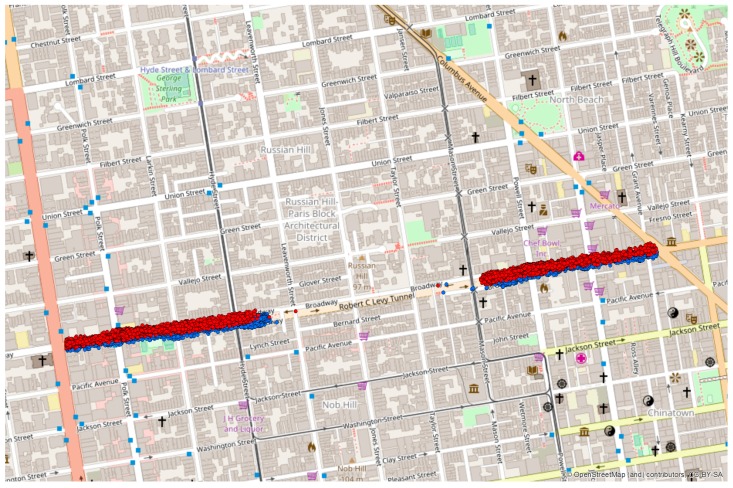
Robert C. Levy (Broadway) Tunnel, São Francisco EUA.
